# A Lost Opportunity to Reduce Future Risk Among Justice-Involved Young Adults Through HIV Testing and Counseling

**DOI:** 10.3390/bs15050578

**Published:** 2025-04-25

**Authors:** Nicholas S. Riano, Jordan Beardslee, Elizabeth Cauffman

**Affiliations:** Department of Psychological Science, School of Social Ecology, University of California, 4220 Social and Behavioral Sciences Gateway Bldg., 214 Pereira Dr, Irvine, CA 92617, USA; jbear@uci.edu (J.B.); cauffman@uci.edu (E.C.)

**Keywords:** legal-system-impacted young adults, HIV testing, HIV risk behavior, HIV testing outcomes

## Abstract

HIV rates among young adults remain high, and those impacted by the justice system are at particular risk. Understanding the factors associated with HIV testing, as well as determining changes in risk behavior after an HIV test, may inform interventions to reduce HIV prevalence among this population. As such, this study sought to determine the individual, contextual, and demographic factors associated with HIV testing among legal-system-impacted young adults and to explore whether a first HIV test is associated with changes in future risk behavior when compared to Never Tested individuals. Significant predictors of HIV testing included the absence of a biological father (OR = 0.68, *p* = 0.049), a higher variety of lifetime offending (OR = 4.74, *p* = 0.015), and living in Philadelphia vs. Phoenix (OR = 3.07, *p* < 0.001). Compared to those never tested for HIV, those newly tested significantly increased in their number of unprotected sexual partners (*b* = 0.52, *p* < 0.001) and in the number of times they had unprotected sex (*b* = 0.47, *p* < 0.001) one year later. This study is one of the first to assess predictors of HIV testing among legal-system-impacted young adults living across both community and carceral settings and to assess changes in risk behavior before and after a first HIV test. Future studies should investigate changes in risk behavior among those newly tested to inform HIV testing and care improvement interventions for this population.

## 1. Introduction

### 1.1. Background/Rationale

In 2022, adolescents and young adults aged 13–24 represented nearly one-fifth of all new HIV diagnoses in the United States (AIDSvu, 2022). Unfortunately, these young people were also some of the least likely to be retained in long-term HIV care or have a well-managed HIV viral load (Centers for Disease Control and Prevention, 2022). Several risk factors contribute to these disparities across individual, contextual, and demographic domains: low rates of HIV testing, a greater number of sexual partners, widespread stigma and socioeconomic challenges, high rates of unprotected sexual intercourse and substance use, and higher prevalence of sexually transmitted diseases (Schnall et al., 2015).

Young adults impacted by the legal system (herein “system-impacted”) represent a population at particular risk for HIV: prevalence in justice populations is between 2 and 5 times higher than the general U.S. population (Office of Juvenile Justice and Delinquency Prevention, 2018; Westergaard et al., 2013). Additionally, prior research demonstrates severe compounded risk in this population: system-impacted young adults report sexual risk behaviors and substance use at rates far greater than the general population (Abram et al., 2017; Welty et al., 2016), are more likely to face HIV risk factors as a result of experiencing neighborhood disorganization, trauma, and exposure to violence (Butcher et al., 2015; Voisin, 2005; Voisin et al., 2011), and are less likely to be formally identified for testing and care by providers (Rhoades et al., 2014; Schnall et al., 2015; Tolou-Shams et al., 2019). While HIV testing in carceral facilities may attenuate certain HIV risks for this population (Haney-Caron et al., 2020), healthcare needs for system-impacted young adults released to community settings after incarceration are frequently overlooked (Belenko et al., 2009; Iroh et al., 2015). As community-based HIV testing has been related to lower sexual risk factors and increased condom use in the general population (Sulat et al., 2018), ensuring knowledge of and access to such services among system-impacted young adults is vital to reduce HIV risk factors and, in so doing, ultimately reduce the prevalence of HIV in this population.

### 1.2. Predictors of HIV Testing

As HIV testing is often touted as the entry to prevention and treatment, ensuring that system-impacted young adults are adequately tested and linked to care is critical for their long-term care (Kurth et al., 2015). However, little is known about the antecedent factors and behaviors associated with HIV testing within aggregate samples of community-based and incarcerated system-impacted young adults. As such, determining the individual, contextual, and demographic factors that are related to HIV testing may elucidate the disproportionate disease burden among this population.

### 1.3. Outcomes of HIV Testing

While a positive HIV diagnosis is related to reduced risk behavior among adults, a negative test result alone does not necessarily preempt a change in subsequent risk behavior (Weinhardt, 2005). Research among college-age adults found that healthcare providers addressing the topic of HIV when discussing health behavior changes and patient self-efficacy can increase rates of HIV testing and reduce high-risk sexual behavior (Rothman et al., 1999). As such, HIV testing coupled with adequate HIV counseling may be associated with an increase in HIV awareness, resulting in a decrease in future risky behavior among community-based system-impacted young adults. However, the extant literature finds that young adults living in the community without prior legal system impact may increase engagement in risky behavior after an HIV test, regardless of the result of the test, especially in the absence of HIV education or counseling (Sen, 2004). As such, it is potentially the coupling of HIV testing with appropriate counseling that may confer benefits among this population, beyond testing alone.

### 1.4. Objectives

Few studies in the current literature examine HIV risk and testing rates among system-impacted young adults, and none to our knowledge have examined how HIV testing may affect future risk behavior and substance use within this population. While past longitudinal research measured HIV risk in young adults post-incarceration (Abram et al., 2017), prior research has not examined the impact of HIV testing on future risk behaviors. As such, the first aim of the current research sought to determine the individual, contextual, and demographic differences between system-impacted young adults who do and do not report HIV testing, and the second aim sought to assess whether receipt of a first HIV test is related to future risky sexual behavior and substance use in the year following the test.

## 2. Materials and Methods

### 2.1. Participants and Setting

Participants were enrolled in Pathways to Desistance, a longitudinal study that followed 1354 juvenile offenders for seven years after an arrest for a serious offense. Eligible youth were recruited from Philadelphia, Pennsylvania, and Phoenix, Arizona, and were enrolled between November 2000 and January 2003. Participants were between 14 and 17 years old at the time of adjudication and had at least one arrest for a felony offense or an arrest for a misdemeanor weapons or sexual assault offense. 

### 2.2. Sampling and Sample Size

The juvenile court systems in Philadelphia, PA, and Maricopa County, AZ, provided information on eligible youth to the study investigators, and all such youth were contacted to participate in the study (*N* = 2009). Before enrollment, all participants provided juvenile assent, and their parents or guardians provided parental consent. Interviews were subsequently conducted in the participant’s home, at a public location in the community, or within a facility if the participant was incarcerated, and were completed regularly over seven years. Recruitment and interviewing protocols were approved by the institutional review boards at all participating universities; see (Schubert et al., 2004) for more information.

### 2.3. Design

#### 2.3.1. Baseline Factors Associated with Future HIV Testing

To determine the individual, contextual, and demographic factors associated with HIV testing among this population, participants were first categorized based on their HIV testing history. This was accomplished through a ‘Lifetime Receipt of HIV Testing’ measure, which asked participants whether they had ever been tested for HIV in their lifetime. This measure was consistently administered to participants beginning at the four-year follow-up of the Pathways study when participants were between the ages of 18 and 23. 

The present study used this lifetime HIV testing measure at two timepoints—the four- and five-year follow-up interviews—to create two HIV testing variables. The first variable, created for Aim 1, measured whether participants were ever tested for HIV in their lifetime prior to the five-year follow-up interview (i.e., participants who reported “yes” to the lifetime HIV test at the four- or five-year interview were coded 1; everyone else was coded 0). This binary lifetime HIV testing variable was created because the primary goal of Aim 1 was to understand the baseline predictors of any HIV testing.

#### 2.3.2. Behavioral Outcomes After HIV Testing

The primary goal of Aim 2 was to understand whether a first HIV test was related to subsequent behavior change, and as such, a more nuanced measure of HIV testing was necessary. Using the HIV test variables measured at the four-year and five-year follow-up interviews, participants were placed into three categories: those who were previously tested for HIV (who reported being tested for HIV in their lifetime at the four-year follow-up interview; “Previously Tested”), those who reported a first HIV test at the five-year follow-up interview (individuals who reported that they had not been tested for HIV in their lifetime at the four-year follow-up, but reported having been tested for HIV at the five-year follow-up interview; “Newly Tested”), and those who reported that they had not been tested for HIV at either the four- or five-year follow-up interviews (“Never Tested”). To strengthen analyses, participants who were missing a response to this question at either the four-year follow-up, the five-year follow-up, or both (*N* = 182) were excluded. Similarly, participants who answered “I don’t know” when asked about whether they had been tested for HIV at the four-year follow-up, the five-year follow-up, or both (*N* = 7) were also excluded. 

To assess whether a new HIV test was related to subsequent high-risk sexual behavior and substance use, the present study compared the behavioral outcomes of those who were newly tested to those who were never tested for HIV in Aim 2. Outcome variables were measured at the six-year follow-up interview, which was approximately one year after the first HIV test for those in the Newly Tested group. The present study also adjusted for prior levels of the outcome variables at the four-year follow-up interview, which was approximately one year prior to the first HIV test for those in the Newly Tested group (and approximately two years before the outcome variable for the Never Tested group). 

### 2.4. Measures, Aim 1: Predictors of HIV Testing

While several factors could influence whether a young person is tested for HIV, the current project sought to accumulate and assess an evidence base of factors associated with HIV testing identified in prior research. For the purposes of this study, intrinsic attributes about a participant’s lived experiences and mental health were considered “individual factors,” attributes associated with the nature of a participant’s living environment and upbringing were considered “contextual factors,” and time-stable attributes were categorized as “demographic factors.” Each factor was measured at the baseline interview of the Pathways study.

#### 2.4.1. Key Grouping Variable: HIV Testing

Participants answered whether they had ever been tested for HIV. As mentioned previously, this measure was collected at the four- and five-year follow-up interviews and was used to create the three key HIV testing groups (previously tested for HIV at some point before the four-year follow-up: Previously Tested, tested for HIV for the first time at the five-year follow-up: Newly Tested, and never tested for HIV by the five-year follow-up: Never Tested). For Aim 1, the Newly Tested and Previously Tested groups were combined into a Lifetime Tested group to examine whether the baseline factors were related to ever having received an HIV test.

#### 2.4.2. Individual Factors

*Mental Health Problem Index.* As prior research has demonstrated that young adults with multiple mental health issues are at increased risk for HIV infection (Auslander et al., 2002), the current study sought to include a measure of mental health symptoms as a predictor of future HIV testing. A Mental Health Problem Index was calculated by summing participant responses from the 53-item Brief Symptom Inventory (Derogatis & Melisaratos, 1983) and dividing the sum by the total number of items endorsed. This resulted in a score that ranged from 0 to 4, with higher scores representing a greater overall severity of symptoms. 

*Number of Sexual Partners.* A higher number of lifetime sexual partners has been well established as a factor that confers additional risk for HIV among young adults (Kann et al., 2018). The present study assessed this factor via a single item, “How many people have you had sex with?”

*Total Exposure to Violence.* Previous research has established a greater exposure to violence as an HIV risk factor, with more exposure conferring more risk (Richardson & Robillard, 2013; Voisin et al., 2011). At baseline, participants responded to an adapted version of the Exposure to Violence Inventory (Selner-O’Hagan et al., 1998), and a total exposure to violence score was calculated by summing the number of violent acts witnessed with the number of violent acts experienced, with higher scores indicating greater exposure to violence.

*Lifetime Non-Marijuana Drug Use.* Substance use is a well-studied risk factor for HIV, especially among young adults impacted by the criminal legal system, such that a greater amount and variety of substance use confers additional risk (Tolou-Shams et al., 2007, 2008, 2019). In the current study, lifetime non-marijuana drug use was measured dichotomously and was calculated as any reported use of sedatives, stimulants, cocaine, opiates, ecstasy, hallucinogens, inhalants, and/or amyl nitrates at the baseline interview. 

*Lifetime Substance Use Treatment.* As previously described, engagement with mental and physical healthcare services is associated with an increased likelihood of HIV testing among young adults (Haney-Caron et al., 2020). As such, the present study sought to determine whether participants had ever engaged in substance use treatment. Participants were asked if they had ever discussed their substance use with a healthcare provider or counselor, attended self-help groups, or were hospitalized due to substance use, and any endorsement was categorized dichotomously.

#### 2.4.3. Contextual Factors

*Biological Father Present in Household.* Prior studies have established the importance of family structure and parental influence in the ability and decision of young people to get tested for HIV (Hadley et al., 2009; Randolph et al., 2017). The presence of a biological father in the home has been previously used as a proxy measure for family structure in developmental samples (Bocknek et al., 2014), and as such, the present study sought to determine whether a biological father was present in participants’ households at baseline. Participants were asked about their family composition, and the presence of a biological father was coded dichotomously.

*Legal System Involvement.* Involvement with the criminal legal system, especially in terms of arrests, has previously been identified as a factor that can increase the risk for HIV among young adults (Tolou-Shams et al., 2007, 2008). In the present study, offense history included the number of times participants had ever been arrested, the age at their first arrest, the variety of offenses they had committed, and whether they had ever been incarcerated. Offense variety included 22 categories of offenses adapted from the Self-Reported Offending scale (Huizinga et al., 1991); offense variety proportion was coded such that scores closer to 1 indicated a greater variety of committed offenses.

#### 2.4.4. Demographic Factors

General demographic information was collected at baseline, including age, study site location, race/ethnicity, and socioeconomic disadvantage. Parental Index of Social Position (ISP) was used as a proxy for socioeconomic disadvantage and was calculated by considering both the highest level of education and current occupation of each parent (Hollingshead, 1957). Of note, this method has been previously validated in both adolescent and adult samples (Galobardes et al., 2007; Lien et al., 2001). Higher scores represented a lower social position, indicating greater socioeconomic disadvantage.

### 2.5. Measures, Aim 2: Outcomes Associated with HIV Testing

The second aim of the study sought to determine whether a first HIV test was related to changes in risky sexual behavior and substance use among system-impacted young adults in the year following the test.

#### 2.5.1. Substance Use Frequency

As substance use frequency has been previously associated with HIV risk behaviors in young adult samples (Patrick et al., 2012; Tolou-Shams et al., 2019), the present study sought to determine the association between a first HIV test and future substance use. At the four- and six-year follow-up interviews, participants self-reported their frequency of alcohol, marijuana, and cigarette use in the past year. A calculated dichotomous measure assessed whether participants had used a non-marijuana drug in the past year.

#### 2.5.2. Risky Sexual Behavior

Risky sexual behavior was also assessed at the four- and six-year follow-up interviews due to its strong association with HIV risk in similar samples (Teplin et al., 2003; Tolou-Shams et al., 2007). Participants reported the number of times they had unprotected sex, as well as their number of unprotected sexual partners in the recall period.

#### 2.5.3. Covariates

All predictor variables included in Aim 1 analyses were included in Aim 2 as covariates, with the addition of time spent out of custody (street time) and age at the four-year follow-up. As the opportunity to engage in risky behavior likely changes while in custody, a measure of the proportion of time participants spent outside of secure facilities during each recall period was calculated for each participant. The number of days spent out of supervised custody (defined as prison, detention, residential, or secure treatment facilities) during each recall period was divided by the total number of days in each recall period. Responses ranged from 0 (participant was confined for the entirety of the recall period) to 1 (participant was out of custody for the entirety of the recall period).

### 2.6. Statistical Analysis, Aim 1: Predictors of HIV Testing

A conditional binary logistic regression model identified the baseline factors associated with lifetime HIV testing (i.e., any HIV testing prior to the five-year follow-up). As we were interested in any HIV testing among this population, the Newly Tested and Previously Tested groups were collapsed into a single category, which enabled distinguishing between Never Tested individuals and those who had received an HIV test at some point in their lives. All previously described individual, contextual, and demographic factors measured at baseline were simultaneously entered into the binary logistic regression predicting the 2-category lifetime HIV test variable.

A supplemental multinomial logistic regression analysis was also conducted using the three-category HIV testing variable (Group 1: Previously tested for HIV at some point before the four-year follow-up, “Previously Tested;” Group 2: HIV tested for the first time at the five-year follow-up, “Newly Tested;” Group 3: Not tested for HIV at any point prior to the five-year follow-up, “Never Tested”) as the outcome. Results from this model are presented in Table S3, which includes the same predictor variables as described above. 

### 2.7. Statistical Analysis, Aim 2: Outcomes Associated with HIV Testing 

Poisson, linear, and logistic regression models determined whether the Newly Tested and Never Tested groups differed on the outcome variables at the six-year follow-up interview, controlling for all predictors in Aim 1, street time, age at the four-year follow-up, and prior levels of the outcome variables (measured at the four-year follow-up). Next, Generalized Estimating Equations examined the difference in the rate of change between the four-year and six-year follow-up interviews for the Newly Tested and Never Tested groups. These models included the same control variables as the prior models, including the predictors from Aim 1, street time, and age at the four-year follow-up. All analyses were conducted with Stata I/C version 16 (StataCorp, 2021).

## 3. Results

### 3.1. Participant Characteristics

Sixty-seven percent of eligible youth agreed to participate in the larger parent study; 1354 were enrolled, and 655 declined to participate. The total sample included both males (*N* = 1170) and females (*N* = 184). The participants were racially and ethnically diverse (44% Black, 29% Latinx, 25% White, and 2% Other Racial Category/Multiracial); these percentages reflect the disproportionate number of youth of color impacted by the criminal legal system (Office of Juvenile Justice and Delinquency Prevention, 2018).

**Figure 1:**
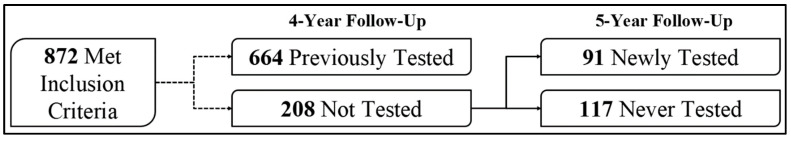
HIV testing categorization.

Due to a small number of females having data on the key HIV testing measure, females (*N* = 184) were dropped, and the final analytic sample contained 872 racially/ethnically diverse male participants (19.7% White, 39.3% Black, 36.4% Latinx, and 4.6% Multiracial/Other Racial Category), who were aged 14–19 years old at baseline (and 18–23 years old at the four-year follow-up). Overall, 664 participants reported being tested for HIV in their lifetime at the four-year follow-up interview, comprising the “Previously Tested” group, 91 participants reported that they had not been tested for HIV in their lifetime at the four-year follow-up but reported having been tested for HIV at the five-year follow-up interview, comprising the “Newly Tested” group, and 117 participants reported that they had not been tested for HIV at both the four- and five-year follow-up interviews, comprising the “Never Tested” group. A visualization of this categorization is presented in Figure 1, descriptive statistics for each predictor variable are presented in Table S1, and correlations between all predictor variables are presented in Table S2.

### 3.2. Aim 1: Predictors of HIV Testing

Aim 1 sought to identify the baseline predictors of lifetime HIV testing. Results from the logistic regression analysis (Table 1) revealed that a greater proportion of offending variety, absence of a biological father in the household, and study site location reported at baseline were significantly associated with lifetime HIV testing. Participants reporting a greater variety of offending behavior were significantly more likely to be tested for HIV (OR 4.74; 95% CI, 1.35–16.66, *p* = 0.015), as were those living in Philadelphia (OR 3.07; 95% CI, 1.89–4.97, *p* < 0.001). Those reporting a biological father present in the household were less likely to have been tested for HIV (OR, 0.68; 95% CI, 0.46–1.00, *p* = 0.049). The mean variance inflation factor (VIF) for included covariates was acceptable at 1.64. Results from the multinomial logistic regression analysis predicting the three-category HIV testing variable (Previously Tested; Newly Tested; Never Tested) revealed similar findings (see Table S3). 

### 3.3. Aim 2: Outcomes Associated with HIV Testing

Aim 2 sought to identify whether young adults’ first HIV test was related to a change in subsequent substance use and risky sexual behavior, and results demonstrated that the Newly Tested group reported a larger number of unprotected sexual partners (*b* = 0.52, *p* < 0.001) and engaged in a greater amount of unprotected sex (*b* = 0.47, *p* < 0.001) than the Never Tested group at the six-year follow-up. Importantly, these results were significant even after controlling for prior levels of the outcome variables, all predictors from Aim 1, street time, and age at the four-year follow-up (Table 2). Note that there were no group differences in alcohol, marijuana, or cigarette use frequency or use of other non-marijuana drugs at the six-year follow-up. Table 2 includes all associations between the covariates and the outcome variables.

**Figure 2:**
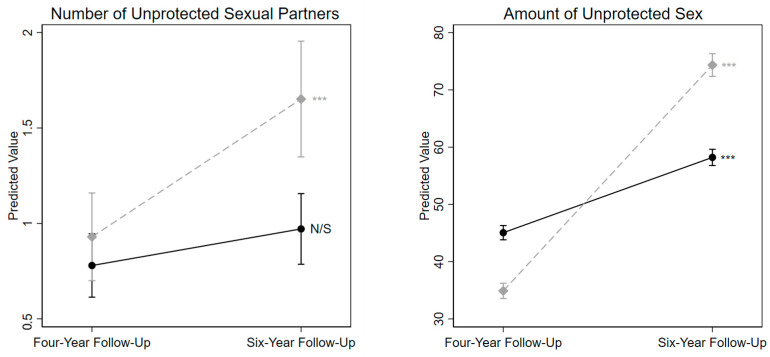
Within-group change on outcome variables. *Notes.* Dashed line represents Newly Tested group, solid line represents Never Tested group. Significance of within-group slopes represented by asterisks and N/S; N/S (not significant), *** *p* < 0.01. For the Newly Tested group, the four-year follow-up value represents the outcome variable one year prior to the first HIV test, and the six-year follow-up value represents the outcome variable approximately one year after the first HIV test. Results from statistical tests comparing the slopes are depicted in Table 3. Predicted values generated from GEE models; only statistically significant/marginally statistically significant outcomes are presented here.

Next, population-averaged Generalized Estimating Equations were used to further evaluate the rate of change for each outcome between the four-year and six-year follow-up and to determine whether the rate of change differed between the Newly Tested and Never Tested groups. As shown in Table 3, the rate of change differed significantly between the two groups on the number of times they had unprotected sex and differed marginally significantly between the two groups on their number of unprotected sexual partners (Table 3, Figure 2). For the amount of unprotected sex, both groups significantly increased between the two timepoints, but the Newly Tested group demonstrated a steeper increase (Newly Tested *b*_TIME_ = 39.44, *p* < 0.001; Never Tested *b*_TIME_ = 13.15, *p* < 0.001). For the number of unprotected sexual partners, the Newly Tested group significantly increased between the two timepoints, while the rate of change between the two timepoints was nonsignificant for the Never Tested group (Newly Tested *b*_TIME_ = 0.72, *p* < 0.001; Never Tested *b*_TIME_ = 0.19, *p* = 0.09). Note that the rate of change between the four- and six-year follow-up interviews did not differ between Newly Tested and Never Tested for any of the other outcomes (consistent with the previous analysis comparing the absolute levels at the six-year follow-up interview).

## 4. Discussion

### 4.1. Baseline Factors Predicting HIV Testing

This is one of the first studies to assess predictors of HIV testing among young adults impacted by the criminal legal system without limiting the sample to only those living in the community or those living in secure custody. Additionally, this study is the first to assess whether HIV testing results in changes in risk behavior one year after the test among young adults impacted by the legal system.

Significant predictors of HIV testing included a greater variety of lifetime offending, the presence of a biological father in the household, and study site location reported at baseline. These results generally follow findings from previous research: for example, increased offending may be indicative of greater integration with judicial systems (probation, court involvement, carceral stays), all of which have been shown to increase HIV testing through mere system contact and its resulting increases in care access, broadly (Tolou-Shams et al., 2015). Thus, the positive association between increased offending and HIV testing demonstrated here is likely a proxy for participants’ larger integration with legal and social services.

Next, it is theoretically plausible that the presence of a biological father in the household would confer a decreased likelihood for HIV testing, as prior research has established this variable as a proxy for overall family structure (Bocknek et al., 2014). As a result, a more structured family may decrease the provider’s necessity to refer a young adult for testing simply due to decreased overall risk for HIV infection. Additionally, as parents have been established as some of their children’s first and most influential HIV/AIDS educators (Krauss & Miller, 2012), it follows that the presence of biological fathers may ultimately reduce the overall risk for HIV. 

Alternatively, it is possible that young male adults who attempt to communicate about sexual risk with their fathers may encounter resistance to HIV testing, as prior studies have demonstrated that masculine norms surrounding HIV and sexual health encourage unprotected sex, reject HIV testing, and discourage seeking help for sexual health issues (Jacques-Aviñó et al., 2019; Marcell et al., 2007). While the Pathways to Desistance study did not explicitly measure sexual risk communication between parents and their children, the presence of a biological father may ultimately reflect parental presence, engagement, and heightened risk monitoring or indicate problematic communication of sexual risk between fathers and sons. However, definitively determining this relationship is beyond the scope of this study, and further research is needed to explore the nuance and interplay between the presence or absence of fathers and subsequent HIV testing.

Study site location also predicted future HIV testing, such that those living in Phoenix at baseline were less likely to be tested by the five-year follow-up than those living in Philadelphia. This may be due to an overall difference in HIV prevalence between Phoenix and Philadelphia: in 2008, around the four- and five-year follow-up period, HIV prevalence in males between the ages of 13 and 24 in Philadelphia County, PA, was nearly six times the rate in Maricopa County, AZ (Centers for Disease Control and Prevention, 2022). As a result, disparities in HIV testing by study site location can likely be geographically explained, such that HIV salience among providers and residents may be higher in places that experience a higher burden of the disease.

It is notable that the presence of several indirect HIV risk factors associated with testing in prior studies (e.g., exposure to violence, mental health symptoms) were not shown to be significantly associated with HIV testing. This may be due to the fact that the present analysis entered all predictors simultaneously—when measured bivariately, these risk factors were significantly associated with testing (data available upon request), but this significance appeared to be accounted for by some of the other more robust predictors.

### 4.2. Behavior Change Between Those Newly and Never Tested for HIV

When compared to those who had never been tested for HIV by the five-year follow-up, those newly tested were more likely to increase the number of times they had unprotected sex in the year after their first HIV test, even after adjusting for all HIV testing predictors identified in Aim 1, age, street time, and prior levels of the outcome variable. In other words, a participant newly tested for HIV increased the number of times they had unprotected sexual intercourse between the year prior and the year after their test when compared to those who had never been tested for HIV. Though research in this area is limited, these findings align with prior work that demonstrated an increase in risk behavior among young adults after an HIV test, regardless of the result of the test, especially in instances where HIV counseling and education were absent (Sen, 2004).

It is possible that this increase in risk behavior after an HIV test is due to a lack of appropriate HIV counseling. Historically, HIV risk-reduction counseling was provided to all patients receiving an HIV test, but this strategy has fallen out of favor due to staffing needs and cost (Farnham et al., 2008). In addition, the advent of the HIV rapid test provides results in minutes, and patients might not be willing to remain at the testing site or clinic to receive such counseling, even if available. As such, test results delivered quickly or via an electronic patient portal may limit the opportunity for counseling. Additionally, this lack of counseling may confer a sense of imperviousness among young adults who receive a negative result, increasing the potential for future risk behavior, a finding that had been demonstrated previously in an adult sample of men who have sex with men (Hoenigl et al., 2015). Regardless, prior studies have demonstrated the effectiveness of risk-reduction counseling in reducing future HIV risk behavior and STI infection (Kamb et al., 1998), and the CDC currently provides sixty evidence-based interventions that have demonstrated effectiveness in reducing future HIV risk (NASTAD, 2022).

While some of these efforts have been demonstrated to be effective in the system-impacted young adult population (Bryan et al., 2009; Donenberg et al., 2015), the majority are differentially available, only offered at specific testing sites, or are not tailored specifically for young adults (Donenberg et al., 2015; Metsch et al., 2013). In order to better reduce future HIV risk within this uniquely vulnerable system-impacted young adult population, carceral facilities, post-release programming, and community clinics serving this population should prioritize making these evidence-based interventions part of the HIV testing process for all patients.

### 4.3. Limitations

This study was limited by several factors. First, all HIV testing information was self-reported by study participants, and independent verification via medical record information was not possible. However, the exclusion of participants who had missing responses or stated that they did not know whether they had been tested likely negates the need to independently verify testing.

Second, while we know who among the participants were tested for HIV and generally when, we do not know the results of these tests, the impetus behind testing administration, nor the place of service. Additionally, as we do not know specifically when those in the Previously Tested group were tested, predictors measured at baseline may have been successive to testing. As such, no conclusions can be made about whether the result of the test influenced any changes in risk behavior or whether facility-administered testing was available. Even still, this knowledge likely would not change the outcomes of the study, as it has been shown that HIV testing can increase future risk behavior regardless of the result (Sen, 2004).

Finally, this study only included male young adults and purposively omitted females due to sample size restrictions. While it is of utmost importance to understand predictors and outcomes of HIV testing for all young adults, both HIV incidence as well as legal system impact is heavily skewed toward males—81% of all new HIV diagnoses in 2018 were among males (US Department of Health and Human Services, 2025), and about 75% of young adults aged 18–24 arrested in 2018 identified as male (Office of Juvenile Justice and Delinquency Prevention, 2018). Nonetheless, future research should examine the research aims studied here with samples of legal-system-impacted young women.

## 5. Conclusions

Young adults impacted by the criminal legal system represent a uniquely vulnerable subpopulation at increased risk for HIV infection, and it is troubling to note the increase in the number of unprotected sexual partners and the amount of unprotected sex after a first HIV test when compared to those who are not tested, above and beyond prior behavior. While it is reassuring to see no significant change in other risk behaviors after an HIV test, future research should examine the behavioral reasons that may underlie increases in risky sexual behavior, namely the number of unprotected sexual partners and the amount of unprotected sex. Specifically, researchers should pay special attention to perceived self-assessment of risk before and after an HIV test, especially when such testing does not include a health education or counseling component. Further, as system-impacted young adults represent a health disparities population by nature of their legal and carceral involvement (Bui et al., 2019), providers and policymakers should increase preventive screening in this population, especially for high-risk infectious diseases like HIV.

Overall, novel interventions specifically tailored to those that endorse the antecedent factors that negatively predict future HIV testing have the potential to improve HIV testing and care among system-impacted young adults, especially when combined with developmentally sensitive approaches to HIV counseling and education. Such efforts may ultimately serve to reduce the increased burden of HIV demonstrated not only within this unique subpopulation of at-risk young adults but also among young adults at large.

## Figures and Tables

**Table 1:** Baseline predictors of any HIV testing vs. no HIV testing (*N* = 872).

	OR	*SE*	*p*	95% CI
*Individual Factors*				
Mental Health Problem Index	1.250	0.175	0.110	0.950, 1.645
Number of Sexual Partners	1.005	0.008	0.479	0.991, 1.020
Total Exposure to Violence	1.043	0.041	0.285	0.966, 1.125
Any Lifetime Non-Marijuana Drug Use	1.102	0.240	0.657	0.718, 1.690
Any Lifetime Substance Use Treatment	1.427	0.362	0.161	0.868, 2.344
*Contextual Factors*				
Biological Father Present	**0.681**	**0.133**	0.049 **	**0.465, 0.998**
Lifetime Number of Arrests	1.056	0.378	0.127	0.985, 1.133
Age at First Arrest	0.918	0.061	0.199	0.806, 1.046
Any Prior Incarceration	1.083	0.228	0.705	0.717, 1.636
Lifetime Offending Variety	**4.735**	**3.039**	0.015 **	**1.346, 16.658**
*Demographic Factors*				
Race/Ethnicity				
White (ref)	*ref*	*ref*	*ref*	*ref*
Black	1.536	0.473	0.164	0.839, 2.801
Latinx	0.806	0.201	0.385	0.494, 1.312
Other Racial Category/Multiracial	0.073	0.312	0.468	0.320, 1.688
Socioeconomic Disadvantage	0.997	0.008	0.683	0.982, 1.012
Age at Baseline	1.028	0.096	0.766	0.857, 1.234
Study Site Location (Philadelphia vs. Phoenix)	**3.066**	**0.757**	<0.001 ***	**1.890, 4.974**

*Notes.* Conditional binary logistic regression predicting any HIV test (i.e., any lifetime testing prior to five-year follow-up interview) vs. no HIV test. All predictor variables were entered simultaneously. Bold typeface indicates statistical significance; ** *p* ≤ 0.05, *** *p* < 0.01.

**Table 2:** Behavioral outcomes one year after HIV testing (i.e., Newly Tested vs. Never Tested), controlling for prior levels of outcome, street time, age, and all Aim 1 predictors.

*Predictors*	Outcome Variables					
	Substance Use Behavior			Risky Sexual Behavior		
	Alcohol Use Frequency (*N* = 197)	Marijuana Use Frequency (*N* = 197)	Cigarette Use Frequency (*N* = 196)	Any Non−Marijuana Drug Use (*N* = 185)	Number of Unprotected Sexual Partners (*N* = 177)	Number of Times Had Unprotected Sex (*N* = 191)
	(B, *p*)	(B, *p*)	(B, *p*)	(B, *p*)	(B, *p*)	(B, *p*)
Newly Tested vs. Never Tested	5.049, 0.239	0.053, 0.574	0.715, 0.235	1.278, 0.630	0.523, 0.001 ***	0.467, <0.001 ***
Prior Level of Outcome Variable	0.403, <0.001 ***	0.161, <0.001 ***	0.569, <0.001 ***	15.812, <0.001 ***	0.171, <0.001 ***	0.005, <0.001 ***
*Individual Factors*						
Mental Health Problem Index	3.190, 0.349	0.042, 0.597	3.902, 0.416	0.560, 0.224	0.318, 0.004 ***	0.166, <0.001 ***
Number of Sexual Partners	−0.042, 0.845	−0.003, 0.567	−0.232, 0.440	1.000, 0.996	0.002, 0.794	0.002, 0.136
Total Exposure to Violence	1.509, 0.098	0.008, 0.708	0.030, 0.982	1.304, 0.041 **	−0.022, 0.519	−0.024, <0.001 ***
Any Lifetime Non-Marijuana Drug Use ^1^	−0.209, 0.967	0.231, 0.039 **	1.780, 0.798	*N/A*	0.321, 0.055	0.674, <0.001 ***
Any Lifetime Substance Use Treatment	7.988, 0.210	−0.030, 0.811	11.258, 0.212	2.227, 0.235	0.290, 0.185	−0.265, <0.001 ***
*Contextual Factors*						
Biological Father Present	0.300, 0.945	0.067, 0.476	7.634, 0.212	1.357, 0.554	−0.087, 0.585	0.084, <0.001 ***
Lifetime Number of Arrests	−0.842, 0.388	−0.042, 0.090	1.049, 0.451	0.779, 0.076	−0.005, 0.880	−0.046, <0.001 ***
Age at First Arrest	−0.730, 0.657	−0.039, 0.224	2.475, 0.292	0.654, 0.031 **	0.017, 0.764	−0.078, <0.001 ***
Any Prior Incarceration	−10.326, 0.041 **	0.109, 0.347	−5.473, 0.436	0.631, 0.478	−0.616, 0.001 ***	0.166, <0.001 ***
Lifetime Offending Variety	7.067, 0.663	0.254, 0.473	25.662, 0.259	42.330, 0.047 **	0.348, 0.529	−0.817, <0.001 ***
*Demographic Factors*						
Race/Ethnicity						
White (ref)	*ref*	*ref*	*ref*	*ref*	*ref*	*ref*
Black	−3.154, 0.695	−0.152, 0.383	−22.170, 0.050 **	0.078, 0.023 **	−0.589, 0.081	−0.240, <0.001 ***
Latinx	−3.976, 0.485	0.102, 0.413	−15.543, 0.052	0.412, 0.174	−0.079, 0.671	0.226, <0.001 ***
Multiracial/Other Racial Category	−13.662, 0.153	−0.394, 0.104	1.594, 0.906	*N/A*	−0.662, 0.095	0.456, <0.001 ***
Socioeconomic Disadvantage	0.112, 0.573	−0.013, 0.004 ***	0.154, 0.582	0.997, 0.890	0.005, 0.428	0.001, 0.143
Concurrent Street Time	11.524, 0.062	0.186, 0.223	17.116, 0.047 **	6.854, 0.056	0.546, 0.040 **	0.865, <0.001 ***
Age at Four-Year Follow-up	−0.938, 0.666	0.095, 0.048 **	−1.784, 0.573	1.477, 0.140	0.180, 0.026 **	−0.049, <0.001 ***
Study Site Location (Philadelphia vs. Phoenix)	−13.672, 0.039 **	0.338, 0.014 **	7.168, 0.441	3.079, 0.134	−0.300, 0.283	0.177, <0.001 ***

*Notes.* Estimates in the table derived from linear, Poisson, or logistic regressions (depending on the distribution of the outcome variable) predicting the behavioral outcome variables that were measured at the six-year follow-up. The primary aim of these analyses was to determine whether the Newly Tested group had worse (or better) behavioral outcomes one year after their first HIV test than the Never Tested group. For those in the Newly Tested group, prior levels of the outcome variables were measured in the one year prior to the first HIV test. As noted in the table, all models controlled for Aim 1 predictors. ^1^ The only exceptions to the inclusion of Aim 1 predictors were that lifetime non-marijuana drug use was not included as a covariate, as prior non-marijuana drug use at the four-year follow-up interview was used instead to be consistent with the other outcomes. Additionally, we added age at the four-year follow-up and street time concurrent to the outcome variable as covariates. Bold typeface indicates statistical significance; ** *p* ≤ 0.05, *** *p* < 0.01.

**Table 3:** Differences in rate of change on outcome variables between Newly Tested and Never Tested HIV groups.

Outcome Variable	Time x Testing Category (i.e., Newly Tested v. Never Tested)		
	B	*p*	95% CI
*Substance Use Behavior*			
Alcohol Use Frequency	6.846	0.110	−1.558, 15.251
Marijuana Use Frequency	−0.006	0.947	−0.185, 0.173
Cigarette Use Frequency	1.814	0.775	−10.613, 14.241
Any Non-Marijuana Drug Use	0.456	0.491	−0.842, 1.754
*Risky Sexual Behavior*			
Number of Unprotected Sexual Partners	*0.355*	*0.059 +*	−0.133, 0.723
Number of Times had Unprotected Sex	**0.500**	<0.001 ***	**0.451, 0.550**

*Notes.* Population-averaged Generalized Estimating Equation (GEE) models predicting the outcome variables (e.g., substance use and risky sexual behavior), controlling for all predictor variables from Aim 1, street time, and age at the four-year follow-up. Estimates in Table 3 represent the interactions between time and the testing variable (Newly Tested vs. Never Tested), which demonstrate whether the rate of change between the four-year follow-up (i.e., prior to HIV test for the Newly Tested group) and the six-year follow-up (i.e., one year after HIV test for the Newly Tested group) differed between the two groups. Bold typeface indicates statistical significance; *** *p* < 0.01, *+* marginally significant.

## Data Availability

All data from the Pathways to Desistance Study are available through the Inter-University Consortium for Political and Social Research (ICPSR 36868).

## Supplementary Materials

The following supporting information can be downloaded at https://www.mdpi.com/article/10.3390/bs15050578/s1, Table S1: Sample characteristics; Table S2: Correlations between Aim 1 predictors; Table S3: Baseline predictors of HIV testing groups (*N* = 872).
