# Supplementary material for: A Lost Opportunity to Reduce Future Risk Among Justice-Involved Young Adults Through HIV Testing and Counseling
Source: Behav Sci (Basel). 2025 Apr 25;15(5):578. doi: 10.3390/bs15050578 (PMC12109017; doi:10.3390/bs15050578)
Supplement: Supplementary file 1 [file behavsci-15-00578-s001.zip › behavsci-3518017-supplementary.pdf]

SUPPLEMENTAL TABLES/FIGURES

Table S1: Sample Characteristics

	Mean	SD	Range	N, %
Individual Factors				
Mental Health Problem Index	1.74	0.68	1-4	872
Number of Sexual Partners	10.39	17.02	0-200	872
Total Exposure to Violence	5.65	2.93	0-13	872
Any Lifetime Non-Marijuana Drug Use				872
No				*452, 52%*
Yes				*420, 48%*
Any Lifetime Substance Use Treatment				872
No				*687, 79%*
Yes				*185, 21%*
Contextual Factors				
Biological Father in Household				872
Absent				*656, 75%*
Present				*216, 25%*
Lifetime Number of Arrests	4.56	4.81	1-40	872
Age at First Arrest	13.75	1.98	5-18	872
Any Prior Incarceration				872
No				*420, 48%*
Yes				*452, 52%*
Lifetime Offending Variety	0.35	0.21	0-0.95	872
Demographic Factors				
Race/Ethnicity				872
White				*172, 20%*
Black				*343, 39%*
Latinx				*317, 36%*
Multiracial/Other Racial Category				*40, 5%*
Socioeconomic Disadvantage	51.56	12.28	11-77	872
Age at Baseline	16.02	1.15	14-19	872
Study Site Location				872
Philadelphia				*449, 51%*
Phoenix				*423, 49%*
Outcome Measure				
HIV Testing Category				872
Previously Tested for HIV				*664, 76%*
Newly Tested for HIV				*91, 10%*
Never Tested for HIV				*117, 13%*

Table S2: Correlations between Aim 1 Predictors

	1	2	3	4	5	6	7	8	9	10	11	12	13	14
1. Mental Health Problem Index	1.00													
2. Num. Sexual Partners	**0.17*****	1.00												
3. Total Exposure to Violence	**0.22*****	**0.25*****	1.00											
4. Any Lifetime Non-Marijuana Drug Use	**0.10*****	0.04	**0.32*****	1.00										
5. Any Lifetime Substance Use Treatment	0.03	0.02	**0.21*****	**0.36*****	1.00									
6. Biological Father Present	-0.03	-0.02	-0.06	0.04	0.01	1.00								
7. Lifetime Num. of Arrests	0.06	**0.11*****	**0.31*****	**0.28*****	**0.26*****	-0.02	1.00							
8. Age at First Arrest	-0.03	-0.03	**-0.18*****	**-0.16*****	**-0.16*****	**0.08****	**-0.52*****	1.00						
9. Any Prior Incarceration	**0.11*****	**0.13*****	**0.23*****	**0.28*****	**0.27*****	**-0.09*****	**0.43*****	**-0.39*****	1.00					
10. Lifetime Offending Variety	**0.17*****	**0.19*****	**0.64*****	**0.51*****	**0.30*****	0.02	**0.46*****	**-0.28*****	**0.35*****	1.00				
11. Race/Ethnicity	**0.08****	0.00	**0.09*****	**0.07****	-0.02	**-0.08****	**0.07****	-0.05	**0.09*****	**0.10*****	1.00			
12. Socioeconomic Disadvantage	0.02	-0.04	-0.04	0.04	0.00	0.02	-0.00	-0.04	**0.09*****	0.02	**0.29*****	1.00		
13. Age at Baseline	0.03	**0.19*****	**0.19*****	**0.14*****	**0.07****	0.00	**0.13*****	**0.30*****	**0.08*****	**0.18*****	0.06	**-0.07****	1.00	
14. Study Site	0.05	**0.24*****	0.06	**-0.32*****	**-0.16*****	**-0.15*****	**-0.08****	0.02	**-0.08****	**-0.18*****	**-0.18*****	**-0.10*****	0.04	1.00

Pairwise correlations of Aim 1 predictor variables. Bold typeface indicates significant correlations. $**p \leq 0.05$, $***p < 0.01$

Table S3: Baseline Predictors of HIV Testing Groups (N=872)

	Main Effect		Newly Vs. Previously Tested		Never Vs. Previously Tested		Newly Vs. Never Tested	
	X^2	*p*	B	*p*	B	*p*	B	*p*
Individual Factors								
Mental Health Problem Index	2.58	0.276						
Number of Sexual Partners	5.05	0.080						
Total Exposure to Violence	1.47	0.479						
Any Lifetime Non-Marijuana Drug Use	1.87	0.393						
Any Lifetime Substance Use Treatment	1.82	0.396						
Contextual Factors								
Biological Father Present	**9.93**	**0.007****	-0.077	0.781	**0.711**	**0.003****	**-0.788**	**0.015****
Lifetime Number of Arrests	2.81	0.245						
Age at First Arrest	2.06	0.358						
Any Prior Incarceration	2.42	0.298						
Lifetime Offending Variety	4.98	0.083						
Demographic Factors								
Race/Ethnicity	6.46	0.374						
Socioeconomic Disadvantage	2.96	0.228						
Age at Baseline	0.25	0.882						
Study Site Location (Philadelphia vs. Phoenix)	**19.99**	**<0.001*****	**-0.851**	**0.008****	**-1.134**	**<0.001*****	0.487	0.257

Notes. Results in table derived from multinomial logistic regression predicting the three category HIV testing group (Group 1: tested for HIV at some point before the four-year follow-up [Previously Tested]; Group 2: tested for the first time at the five-year follow-up [Newly Tested]; Group 3: not tested for HIV any time before the five-year follow-up interview [Never Tested]). All covariates included simultaneously.
**$p \leq 0.05$, ***$p < 0.01$
